# The Chevrel phase HgMo_6_S_8_

**DOI:** 10.1107/S1600536809012495

**Published:** 2009-04-18

**Authors:** Diala Salloum, Patrick Gougeon, Michel Potel

**Affiliations:** a Laboratoire de Chimie du Solide et Inorganique Moléculaire, URA CNRS No. 6511, Université de Rennes I, Avenue du Général Leclerc, 35042 Rennes CEDEX, France

## Abstract

The crystal structure of HgMo_6_S_8_, mercury(II) hexa­molybdenum octa­sulfide, is based on (Mo_6_S_8_)S_6_ cluster units (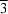 symmetry) inter­connected through inter­unit Mo—S bonds. The Hg^2+^ cations occupy large voids between the different cluster units and are covalently bonded to two S atoms. The Hg atoms and one S atom lie on sites with crystallographic 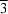 and 3 symmetry, respectively. Refinement of the occupancy factor of the Hg atom led to the composition Hg_0.973 (3)_Mo_6_S_8_.

## Related literature

For isotypic structures, see: Chevrel & Sergent (1982 ▶). For a previous report on the title compound as a polycrystalline material, see: Tarascon *et al.* (1983 ▶). For crystallographic background, see: Becker & Coppens (1974 ▶); Johnson & Levy (1974 ▶).

## Experimental

### 

#### Crystal data

Hg_0.973_Mo_6_S_8_
*M* *_r_* = 1027.3
Trigonal, 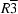

*a* = 9.4319 (3) Å
*c* = 10.7028 (3) Å
*V* = 824.57 (4) Å^3^
*Z* = 3
Mo *K*α radiation
μ = 21.62 mm^−1^
*T* = 293 K
0.08 × 0.07 × 0.06 mm

#### Data collection

Nonius KappaCCD diffractometer
Absorption correction: analytical (de Meulenaer & Tompa, 1965 ▶) *T*_min_ = 0.298, *T*_max_ = 0.384
5784 measured reflections
1121 independent reflections
1069 reflections with *I* > 2σ(*I*)
*R*_int_ = 0.044

#### Refinement

*R*[*F* ^2^ > 2σ(*F* ^2^)] = 0.025
*wR*(*F* ^2^) = 0.026
*S* = 1.74
1121 reflections
31 parameters
Δρ_max_ = 2.64 e Å^−3^
Δρ_min_ = −1.57 e Å^−3^

### 

Data collection: *COLLECT* (Nonius, 1998 ▶); cell refinement: *COLLECT*; data reduction: *EVALCCD* (Duisenberg *et al.*, 2003 ▶); program(s) used to solve structure: *SIR97* (Altomare *et al.*, 1999 ▶); program(s) used to refine structure: *JANA2000* (Petříček & Dušek, 2000 ▶); molecular graphics: *DIAMOND* (Bergerhoff, 1996 ▶); software used to prepare material for publication: *JANA2000*.

## Supplementary Material

Crystal structure: contains datablocks global, I. DOI: 10.1107/S1600536809012495/wm2226sup1.cif

Structure factors: contains datablocks I. DOI: 10.1107/S1600536809012495/wm2226Isup2.hkl

Additional supplementary materials:  crystallographic information; 3D view; checkCIF report

## Figures and Tables

**Table 1:** Selected bond lengths (Å)

Hg1—S1	2.3914 (8)
Mo1—Mo1^i^	2.7184 (3)
Mo1—Mo1^ii^	2.7515 (3)
Mo1—S1	2.4108 (7)
Mo1—S2	2.4236 (6)
Mo1—S2^iii^	2.4896 (8)
Mo1—S2^ii^	2.4933 (6)
Mo1—S2^iv^	2.4340 (8)

Symmetry codes: (i) 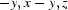; (ii) 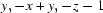; (iii) 
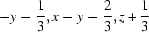
; (iv) 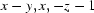.

**Fig. 1.**
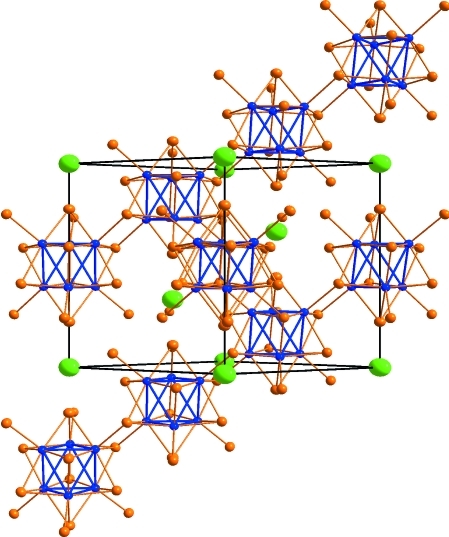
: View of HgMo6S8 along [110].

**Fig. 2.**
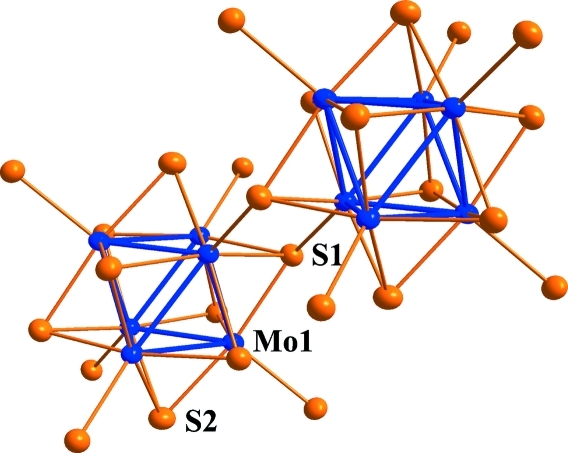
: Plot showing the atom-numbering scheme and the interunit linkage of the (Mo6S8)S6 cluster units. Displacement ellipsoids are drawn at the 97% probability level.

## supplementary crystallographic information

### Comment

The superconducting compound HgMo_6_S_8_ was first synthesized as a powder sample by Tarascon *et al.* (1983), but no details were given on its crystal structure. In the present study, we present the crystal structure refinement of HgMo_6_S_8_ that has been determined from single-crystal X-ray diffraction data. The title compound is isostructural with the hexagonal Chevrel phases *M*Mo_6_*X*_8_ where *M* is a large cation (*M* = alkali metal, alkaline earth, lanthanide, actinide *etc*.; *X* = S, Se, Te) [see, for instance, Chevrel & Sergent (1982)]. As a consequence its crystal structure consists of octahedral Mo_6_ clusters surrounded by fourteen sulfur atoms with eight of them forming a distorted cube (*i*-type ligands) and the remaining six capping the faces of the S_8_ cube (*a*-type ligands). In the structure of HgMo_6_S_8_, a part of the chalcogen atoms of the Mo_6_S*^i^*_8_S*^a^*_6_ unit are shared according to the formula Mo_6_S*^i^*_2_S*^i-a^*_6/2_S*^a-i^*_6/2_ to form the three-dimensional Mo—S network. The Mo_6_S_8_ cluster unit is centered at Wyckoff position 6*b* (3 symmetry). The Mo—Mo distances within the Mo_6_ clusters are 2.7184 (3) Å for the intra-triangle distances (distances within the Mo_3_ triangles formed by the Mo atoms related through the threefold axis) and 2.7515 (3) Å for the inter-triangle distances. Each Mo atom is surrounded by five S atoms (4 S1 and 1 S2) forming a distorted square-based pyramid. The apex of the pyramid is shared with an adjacent unit and thus ensures the three-dimensional cohesion. Consequently, each Mo_6_S_8_ unit is interconnected to 6 Mo_6_S_8_ units to form the Mo—S framework. It results from this arrangement that the shortest intercluster Mo1—Mo1 distances between the Mo_6_ clusters is 3.2934 (3) Å, indicating only weak metal-metal interaction. The Hg^2+^ cations reside in the large eight-coordinate voids formed by the chalcogen atoms from eight different Mo_6_S_8_ units. They are covalently bonded to two S2 atoms at a distance of 2.3914 (8) Å.

HgMo_6_S_8_ was found to be superconducting at 8 K from DC-susceptibility measurements on a batch of single crystals.

### Experimental

HgMo_6_S_8_ was obtained in three steps involving, first, the syntheses of single-crystal of InMo_6_S_8_ by solid state reaction, then the preparation of the binary compound Mo_6_S_8_ by 'chimie douce' methods and, finally, the synthesis of the title compound by inserting mercury into the Mo_6_S_8_ host structure at low temperatures. Single crystals of InMo_6_S_8_ were obtained from a stoichiometric mixture of In_2_S_3_, MoS_2_ and Mo. All handlings of materials were done in an argon-filled glove box. The initial mixture (*ca* 5 g) was cold pressed and loaded into a molybdenum crucible, which was sealed under a low argon pressure using an arc-welding system. The charge was heated at the rate of 300 K/h up to 1773 K, the temperature which was held for six hours, then cooled at 100 K/h down to 1273 K and finally furnace cooled. Mo_6_S_8_ was obtained by oxidation of single-crystals of InMo_6_S_8_ by iodine in a glass tube sealed under vacuum. The end of the tube containing the crystals of the In compound and an excess of iodine was placed in a furnace with about 3 cm of the other end sticking out of the furnace, at about room temperature. The furnace was then heated at 523 K for 96 h. At the end of the reaction, crystals of InI_3_ and I_2_ were obtained at the cooler end of the tube. Finally, HgMo_6_S_8_ was prepared by diffusion of mercury into crystals of Mo_6_S_8_ in a silica glass tube sealed under vacuum at 673 K during 96 h.

### Refinement

The structure was refined using an anisotropic approximation and converged at an reliability factor *R*(*F*) = 0.034. Analyses of the difference Fourier maps revealed positive and negative residual peaks around the Hg atom. Fourth-order tensors in the Gram-Charlier expansion (Johnson & Levy, 1974) of the mercury displacement factor were used to describe the electron density around this site. The resulting *R* value dropped to 0.025 for only five additional parameters. Refinement of the occupancy factor of the Hg atom led to the final composition Hg_0.973 (3)_Mo_6_S_8_.

### Crystal data

**Table:**

Hg_0.973_Mo_6_S_8_	*D*_x_ = 6.204 (1) Mg m^−^^3^
*M**_r_* = 1027.3	Mo *K*α radiation, λ = 0.71069 Å
Trigonal, *R*3	Cell parameters from 7043 reflections
Hall symbol: -R 3	θ = 2.0–42.1°
*a* = 9.4319 (3) Å	µ = 21.62 mm^−^^1^
*c* = 10.7028 (3) Å	*T* = 293 K
*V* = 824.57 (4) Å^3^	Truncated cube, black
*Z* = 3	0.08 × 0.07 × 0.06 mm
*F*(000) = 1374	

### Data collection

**Table:**

Nonius KappaCCD diffractometer	1121 independent reflections
Radiation source: fine-focus sealed tube	1069 reflections with *I* > 2σ(*I*)
horizontally mounted graphite crystal	*R*_int_ = 0.044
Detector resolution: 9 pixels mm^-1^	θ_max_ = 39.8°, θ_min_ = 3.1°
ω– and φ–scans	*h* = −16→16
Absorption correction: analytical (de Meulenaer & Tompa, 1965)	*k* = −16→16
*T*_min_ = 0.298, *T*_max_ = 0.384	*l* = −13→19
5784 measured reflections	

### Refinement

**Table:**

Refinement on *F*	Weighting scheme based on measured s.u.'s *w* = 1/σ^2^(*F*)
*R*[*F*^2^ > 2σ(*F*^2^)] = 0.025	(Δ/σ)_max_ = 0.001
*wR*(*F*^2^) = 0.026	Δρ_max_ = 2.64 e Å^−^^3^
*S* = 1.74	Δρ_min_ = −1.57 e Å^−^^3^
1121 reflections	Extinction correction: B-C type 1 Lorentzian isotropic (Becker & Coppens, 1974)
31 parameters	Extinction coefficient: 0.020681

### Fractional atomic coordinates and isotropic or equivalent isotropic displacement parameters (Å^2^)

**Table:**

	*x*	*y*	*z*	*U*_iso_*/*U*_eq_	Occ. (<1)
Hg1	0	0	0	0.0339 (4)	0.973 (3)
Mo1	−0.01555 (2)	−0.17363 (2)	−0.394419 (15)	0.00748 (7)	
S1	0	0	−0.22344 (8)	0.0113 (2)	
S2	−0.03460 (6)	−0.31591 (7)	−0.58775 (4)	0.00933 (17)	

### Atomic displacement parameters (Å^2^)

**Table:**

	*U*^11^	*U*^22^	*U*^33^	*U*^12^	*U*^13^	*U*^23^
Hg1	0.0384 (4)	0.0384 (4)	0.0249 (6)	0.0192 (2)	0	0
Mo1	0.00780 (9)	0.00831 (9)	0.00617 (10)	0.00391 (6)	0.00003 (5)	−0.00036 (5)
S1	0.0126 (2)	0.0126 (2)	0.0088 (3)	0.00628 (12)	0	0
S2	0.0097 (2)	0.0096 (2)	0.0087 (2)	0.00476 (17)	0.00067 (15)	−0.00032 (15)

### Geometric parameters (Å, °)

**Table:**

Hg1—S1	2.3914 (8)	Mo1—Mo1^ix^	2.7184 (3)
Hg1—S1^i^	2.3914 (8)	Mo1—Mo1^x^	2.7515 (3)
Hg1—S2^ii^	3.2056 (4)	Mo1—Mo1^xi^	2.7184 (4)
Hg1—S2^iii^	3.2056 (4)	Mo1—Mo1^xii^	2.7515 (2)
Hg1—S2^iv^	3.2056 (7)	Mo1—S1	2.4108 (7)
Hg1—S2^v^	3.2056 (7)	Mo1—S2	2.4236 (6)
Hg1—S2^vi^	3.2056 (8)	Mo1—S2^xiii^	2.4896 (8)
Hg1—S2^vii^	3.2056 (8)	Mo1—S2^x^	2.4933 (6)
Mo1—Mo1^viii^	3.8679 (3)	Mo1—S2^xii^	2.4340 (8)
Mo1—Mo1^iii^	3.2131 (2)		
			
S1—Hg1—S1^i^	180	Mo1^x^—Mo1—Mo1^iii^	97.693 (7)
S1—Hg1—S2^ii^	105.278 (8)	Mo1^x^—Mo1—Mo1^ix^	90
S1—Hg1—S2^iii^	74.722 (8)	Mo1^x^—Mo1—Mo1^xi^	60.398 (8)
S1—Hg1—S2^iv^	105.278 (9)	Mo1^x^—Mo1—Mo1^xii^	59.205 (7)
S1—Hg1—S2^v^	74.722 (9)	Mo1^x^—Mo1—S1	115.964 (15)
S1—Hg1—S2^vi^	105.278 (9)	Mo1^x^—Mo1—S2	55.677 (18)
S1—Hg1—S2^vii^	74.722 (9)	Mo1^x^—Mo1—S2^xiii^	138.626 (14)
S1^i^—Hg1—S1	180	Mo1^x^—Mo1—S2^x^	54.776 (13)
S1^i^—Hg1—S2^ii^	74.722 (8)	Mo1^x^—Mo1—S2^xii^	114.515 (14)
S1^i^—Hg1—S2^iii^	105.278 (8)	Mo1^xi^—Mo1—Mo1^iii^	96.739 (8)
S1^i^—Hg1—S2^iv^	74.722 (9)	Mo1^xi^—Mo1—Mo1^ix^	60.000 (8)
S1^i^—Hg1—S2^v^	105.278 (9)	Mo1^xi^—Mo1—Mo1^x^	60.398 (8)
S1^i^—Hg1—S2^vi^	74.722 (9)	Mo1^xi^—Mo1—Mo1^xii^	90
S1^i^—Hg1—S2^vii^	105.278 (9)	Mo1^xi^—Mo1—S1	55.682 (12)
S2^ii^—Hg1—S2^iii^	180	Mo1^xi^—Mo1—S2	116.065 (18)
S2^ii^—Hg1—S2^iv^	113.319 (18)	Mo1^xi^—Mo1—S2^xiii^	135.971 (18)
S2^ii^—Hg1—S2^v^	66.681 (18)	Mo1^xi^—Mo1—S2^x^	55.48 (2)
S2^ii^—Hg1—S2^vi^	113.319 (17)	Mo1^xi^—Mo1—S2^xii^	117.362 (19)
S2^ii^—Hg1—S2^vii^	66.681 (17)	Mo1^xii^—Mo1—Mo1^iii^	148.317 (7)
S2^iii^—Hg1—S2^ii^	180	Mo1^xii^—Mo1—Mo1^ix^	60.398 (6)
S2^iii^—Hg1—S2^iv^	66.681 (18)	Mo1^xii^—Mo1—Mo1^x^	59.205 (7)
S2^iii^—Hg1—S2^v^	113.319 (18)	Mo1^xii^—Mo1—Mo1^xi^	90
S2^iii^—Hg1—S2^vi^	66.681 (17)	Mo1^xii^—Mo1—S1	115.964 (13)
S2^iii^—Hg1—S2^vii^	113.319 (17)	Mo1^xii^—Mo1—S2	57.184 (12)
S2^iv^—Hg1—S2^ii^	113.319 (18)	Mo1^xii^—Mo1—S2^xiii^	133.837 (19)
S2^iv^—Hg1—S2^iii^	66.681 (18)	Mo1^xii^—Mo1—S2^x^	113.894 (15)
S2^iv^—Hg1—S2^v^	180	Mo1^xii^—Mo1—S2^xii^	55.318 (14)
S2^iv^—Hg1—S2^vi^	113.319 (19)	S1—Mo1—S2	170.65 (2)
S2^iv^—Hg1—S2^vii^	66.681 (19)	S1—Mo1—S2^xiii^	93.53 (2)
S2^v^—Hg1—S2^ii^	66.681 (18)	S1—Mo1—S2^x^	90.323 (17)
S2^v^—Hg1—S2^iii^	113.319 (18)	S1—Mo1—S2^xii^	91.758 (14)
S2^v^—Hg1—S2^iv^	180	S2—Mo1—S2^xiii^	95.79 (2)
S2^v^—Hg1—S2^vi^	66.681 (19)	S2—Mo1—S2^x^	87.39 (2)
S2^v^—Hg1—S2^vii^	113.319 (19)	S2—Mo1—S2^xii^	88.750 (19)
S2^vi^—Hg1—S2^ii^	113.319 (17)	S2^xiii^—Mo1—S2	95.79 (2)
S2^vi^—Hg1—S2^iii^	66.681 (17)	S2^xiii^—Mo1—S2^x^	99.70 (2)
S2^vi^—Hg1—S2^iv^	113.319 (19)	S2^xiii^—Mo1—S2^xii^	91.39 (2)
S2^vi^—Hg1—S2^v^	66.681 (19)	S2^x^—Mo1—S2	87.39 (2)
S2^vi^—Hg1—S2^vii^	180	S2^x^—Mo1—S2^xiii^	99.70 (2)
S2^vii^—Hg1—S2^ii^	66.681 (17)	S2^x^—Mo1—S2^xii^	168.58 (2)
S2^vii^—Hg1—S2^iii^	113.319 (17)	S2^xii^—Mo1—S2	88.750 (19)
S2^vii^—Hg1—S2^iv^	66.681 (19)	S2^xii^—Mo1—S2^xiii^	91.39 (2)
S2^vii^—Hg1—S2^v^	113.319 (19)	S2^xii^—Mo1—S2^x^	168.58 (2)
S2^vii^—Hg1—S2^vi^	180	Hg1—S1—Mo1	139.382 (14)
Mo1^viii^—Mo1—Mo1^iii^	133.459 (8)	Hg1—S1—Mo1^ix^	139.382 (13)
Mo1^viii^—Mo1—S1	85.136 (14)	Hg1—S1—Mo1^xi^	139.382 (14)
Mo1^viii^—Mo1—S2	85.600 (16)	Mo1—S1—Mo1^ix^	68.64 (2)
Mo1^viii^—Mo1—S2^xiii^	176.394 (13)	Mo1—S1—Mo1^xi^	68.64 (2)
Mo1^viii^—Mo1—S2^x^	83.677 (18)	Mo1^ix^—S1—Mo1	68.64 (2)
Mo1^viii^—Mo1—S2^xii^	85.310 (16)	Mo1^ix^—S1—Mo1^xi^	68.64 (2)
Mo1^iii^—Mo1—Mo1^viii^	133.459 (8)	Mo1^xi^—S1—Mo1	68.64 (2)
Mo1^iii^—Mo1—Mo1^ix^	147.479 (10)	Mo1^xi^—S1—Mo1^ix^	68.64 (2)
Mo1^iii^—Mo1—Mo1^x^	97.693 (7)	Hg1^xiv^—S2—Mo1	125.450 (18)
Mo1^iii^—Mo1—Mo1^xi^	96.739 (8)	Hg1^xiv^—S2—Mo1^x^	98.407 (18)
Mo1^iii^—Mo1—Mo1^xii^	148.317 (7)	Hg1^xiv^—S2—Mo1^xv^	97.225 (18)
Mo1^iii^—Mo1—S1	92.988 (11)	Hg1^xiv^—S2—Mo1^xii^	156.59 (2)
Mo1^iii^—Mo1—S2	92.457 (12)	Mo1—S2—Mo1^x^	69.005 (19)
Mo1^iii^—Mo1—S2^xiii^	49.898 (13)	Mo1—S2—Mo1^xv^	132.74 (2)
Mo1^iii^—Mo1—S2^x^	49.797 (18)	Mo1—S2—Mo1^xii^	68.041 (15)
Mo1^iii^—Mo1—S2^xii^	141.203 (18)	Mo1^x^—S2—Mo1	69.005 (19)
Mo1^ix^—Mo1—Mo1^iii^	147.479 (10)	Mo1^x^—S2—Mo1^xv^	129.09 (2)
Mo1^ix^—Mo1—Mo1^x^	90	Mo1^x^—S2—Mo1^xii^	66.955 (19)
Mo1^ix^—Mo1—Mo1^xi^	60.000 (8)	Mo1^xv^—S2—Mo1	132.74 (2)
Mo1^ix^—Mo1—Mo1^xii^	60.398 (6)	Mo1^xv^—S2—Mo1^x^	129.09 (2)
Mo1^ix^—Mo1—S1	55.682 (11)	Mo1^xv^—S2—Mo1^xii^	80.305 (15)
Mo1^ix^—Mo1—S2	117.489 (13)	Mo1^xii^—S2—Mo1	68.041 (15)
Mo1^ix^—Mo1—S2^xiii^	131.337 (14)	Mo1^xii^—S2—Mo1^x^	66.955 (19)
Mo1^ix^—Mo1—S2^x^	115.28 (2)	Mo1^xii^—S2—Mo1^xv^	80.305 (15)
Mo1^ix^—Mo1—S2^xii^	57.566 (18)		

Symmetry codes: (i) −*x*, −*y*, −*z*; (ii) *x*+1/3, *y*+2/3, *z*+2/3; (iii) −*x*−1/3, −*y*−2/3, −*z*−2/3; (iv) −*y*−2/3, *x*−*y*−1/3, *z*+2/3; (v) *y*+2/3, −*x*+*y*+1/3, −*z*−2/3; (vi) −*x*+*y*+1/3, −*x*−1/3, *z*+2/3; (vii) *x*−*y*−1/3, *x*+1/3, −*z*−2/3; (viii) −*x*, −*y*, −*z*−1; (ix) −*y*, *x*−*y*, *z*; (x) *y*, −*x*+*y*, −*z*−1; (xi) −*x*+*y*, −*x*, *z*; (xii) *x*−*y*, *x*, −*z*−1; (xiii) −*y*−1/3, *x*−*y*−2/3, *z*+1/3; (xiv) *x*−1/3, *y*−2/3, *z*−2/3; (xv) −*x*+*y*+1/3, −*x*−1/3, *z*−1/3.
